# Understanding Personalized Dynamics in Eating Disorders: A Dynamic Time Warp Analysis

**DOI:** 10.1002/eat.24407

**Published:** 2025-03-13

**Authors:** A. E. Dingemans, E. J. Giltay, P. J. Rohrbach, E. F. van Furth, M. C. T. Slof‐Op ’t Landt

**Affiliations:** ^1^ Rivierduinen Eating Disorders Ursula Leiden the Netherlands; ^2^ Department of Psychiatry Leiden University Medical Center Leiden the Netherlands; ^3^ Health Campus the Hague, Department of Public Health & Primary Care Leiden University Medical Center Leiden the Netherlands; ^4^ Faculty of Psychology, Department of Clinical Psychology Open Universiteit Heerlen the Netherlands

**Keywords:** anxiety, dynamic time warp, eating disorders, E‐health, help‐seeking behavior, laxatives abuse, low‐threshold interventions, social support, symptom interaction, temporal relationships

## Abstract

**Objective:**

To enhance our understanding of the processes of change and the interaction of symptoms, we applied a relatively novel method known as Dynamic Time Warp to data from low‐threshold internet‐based interventions directed at decreasing eating disorder (ED) symptoms and increasing help‐seeking.

**Method:**

Utilizing data from the Featback study, we examined how various factors such as ED psychopathology, binge eating, vomiting, laxative use, BMI, anxiety, depression, self‐efficacy, social support, well‐being, and health‐related quality of life interplayed over a period of 14 months among 355 individuals at six different time points. Moreover, we explored which symptoms exerted a significant temporal relationship on others (with high out‐strength) and which were most affected by other symptoms (with high in‐strength).

**Results:**

The sample included participants with different types of ED symptoms and high levels of severity. On a group level, we identified four dimensions with similar within‐person trajectories: (1) Depression, anxiety, ED psychopathology, health‐related quality of life, and self‐rated health; (2) binge eating and vomiting; (3) self‐efficacy and social support; (4) BMI, well‐being, and laxative use. Directed analyses revealed that social support and anxiety had the highest significant out‐strength (i.e., temporal lead), indicating their changes preceded those of other factors, while laxative use and well‐being were among those that mostly lagged behind those of other items (with significant in‐strength).

**Discussion:**

Depressive and anxiety symptom severity were strongly connected to ED severity. Social support may be an important factor to address first as it may drive other factors and symptoms in patients with EDs.

**Summary**

- Our study used a novel method, Dynamic Time Warp, to analyze symptom changes over 14 months in individuals undergoing online support for eating disorders (EDs).
- We found that social support and anxiety led to changes in other symptoms, while well‐being and laxative use followed changes.
- This suggests that enhancing social support could be a key strategy in improving ED symptoms and overall mental health.

## 1 Introduction

Eating disorders (EDs) are psychiatric disorders characterized by high rates of comorbidity, chronicity, relapse, and mortality (Keel and Brown 2010; Smink et al. 2013). The course and outcome of EDs vary depending on the type and severity of the ED, but, in general, can be regarded as rather unsatisfactory. On average, only 50% of those who seek help will make a full recovery (Treasure et al. 2020). The chronic and persistent nature of EDs may be driven by ongoing interactions between their different components over time. To understand these interactions thoroughly, researchers need panel data (i.e., time series) that track individual patients over a period. This type of data enables a detailed study of within‐person dynamics, focusing on how factors change and interact within a single person, rather than averaging observations across a group. By identifying components that consistently occur before others, researchers can uncover patterns that strengthen the network of interconnected factors driving the disorder. Understanding these patterns could reveal plausible treatment targets for more effective treatment strategies.

Two previous randomized controlled trials (RCTs) (Aardoom et al. 2016a; Rohrbach, Dingemans, Spinhoven, et al. 2022) showed that a fully automated, easily accessible internet‐based self‐help program for EDs (“Featback”), weekly chat or email support from an expert patient (or psychologist), or the combination of both, was effective in reducing ED symptoms compared to a waiting list control condition. Featback consists of psychoeducation and a fully automated self‐monitoring and feedback system. After completing a monitoring questionnaire, participants received a supportive feedback message that matched the participant's answers. Self‐monitoring is an important clinical technique and helps to gain a more comprehensive understanding of one's psychopathology (Cohen et al. 2013). Involving individuals with a lived experience of an ED, often called expert patients, may help to overcome shame and anxiety, promote acceptance of help from others, and reduce the gap between symptom onset and treatment. Their experience provides a self‐evident credibility to patients, which may increase feelings of empowerment in individuals with acute ED symptoms, thus improving their self‐efficacy and self‐management skills (Rohrbach, Dingemans, Spinhoven, et al. 2022). Low‐threshold interventions for EDs may also stimulate help‐seeking behaviors (Rohrbach, Dingemans, Spinhoven, et al. 2022). Individual trajectories of recovery, however, may differ considerably between patients (De Vos et al. 2023): not every individual will show a similar response to or benefit from an intervention. For example, motivation to change, interpersonal functioning, psychiatric comorbidity, and the specific presentation of ED symptoms have been found to influence treatment outcomes for EDs, either as predictors or moderators (Kass et al. 2014; Lydecker and Grilo 2022; Vall and Wade 2015; Volker et al. 2014). Several reviews showed that EDs and other mental health problems such as depression and anxiety are highly associated (Tan et al. 2023). For example, ED psychopathology is one of the risk factors for depression and vice versa (Puccio et al. 2016), and there is evidence to suggest that the relationship between anxiety and anorexia nervosa (AN) can be bi‐directional (Lloyd et al. 2019). Research regarding predictors and moderators of treatment outcomes for Featback specifically has also been conducted. The findings of two Featback studies were non‐overlapping (Aardoom et al. 2017; Rohrbach, Fokkema, et al. 2023); one analysis suggested that baseline social support may serve as a predictor (Rohrbach, Fokkema, et al. 2023), while the other indicated that prior manifestation of ED symptoms could act as a moderator (Aardoom et al. 2017) in changes to ED psychopathology.

Another approach to increase our understanding of the process of ED recovery would be to further examine changes and heterogeneity in individual trajectories. There are various methods, such as multilevel Vector Autoregression (mlVAR), for conducting prospective network analyses. mlVAR estimates causal relationships through lagged connections, relying on assumptions like stationarity and conditioning on all variables. However, stationarity is challenging to uphold in patients undergoing treatment (Bringmann 2024). A relatively new approach, the dynamic time warp (DTW), could be used to give insight into these complex dynamics over time (Hebbrecht et al. 2020). In contrast with other methods, DTW identifies co‐occurrence and synchrony in time‐series data and examines pairwise relationships independently, making it a non‐multivariate method. Comparable to network theory (Borsboom and Cramer 2013), DTW conceptualizes symptoms as mutually interacting, often reciprocally reinforcing, elements of a complex network. DTW aims to find longitudinal co‐occurring patterns among symptoms by comparing and aligning time series data at the individual level. These co‐occurring patterns are subsequently aggregated at the group level to identify clusters of symptoms that show similar change patterns over time. In addition, the temporal relationships between the symptoms (i.e., which symptom changes precede similar changes in others or vice versa) can be examined by a directed (lag‐1) approach of DTW (Mesbah et al. 2024).

There have been three previous DTW studies among ED patients (Kopland et al. 2025; Kopland et al. 2024; Slof‐Op 't Landt et al. 2022). In the first study of a mixed ED sample of 255 individuals assessed at four time points (Slof‐Op 't Landt et al. 2022), four robust dimensions of ED psychopathology symptoms were found that appeared to change together over time in this large naturalistic sample of patients with an ED; ‘restraint/rules', ‘worries and preoccupation’, ‘secret eating/fasting’, and ‘weight and shape concerns'. The latter dimension consisted of shape and weight related concerns, like fear of weight gain, shape and weight satisfaction, and the desire to lose weight. In the second study in which a mixed ED sample of 122 individuals in inpatient treatment assessed at 14 time points was examined (Kopland et al. 2024), the network revealed three robust clusters of symptoms over time: (1) ED behavior, (2) inhibition, and (3) cognitions and feelings about body and weight. Overvaluation of shape had the highest out‐strength, indicating that it generally preceded and predicted changes in another symptom. In a subsequent study by Kopland et al. (2025), the dynamics between the three ED clusters, self‐compassion, anxiety and depression symptoms were examined in the same sample with at least four assessments in time. Hopelessness had the highest out‐strength, while ED behavior, emotional distress tolerance, and self‐kindness had high in‐strength. Other longitudinal network studies in the ED field showed that symptoms related to weight and shape also appear to be the most central symptoms at the group level (Levinson et al. 2018, 2020, 2022) and may therefore form the core psychopathology that maintains the disorder. However, almost all the longitudinal network or DTW studies have only taken ED symptoms into account. Monteleone and Cascino (2021) suggested that the overvaluation of shape and weight forms the core of all ED psychopathology and is hypothesized to maintain the ED, but other symptoms and attributes like anxiety, depression, health, well‐being, and healthy behaviors should not be neglected. The interplay between these variables is a complex and interconnected web that profoundly influences an individual's journey toward recovery. Understanding how these factors interact can provide valuable insights for healthcare professionals, caregivers, and individuals with an ED themselves. Data from the second Featback RCT (Rohrbach, Dingemans, Spinhoven, et al. 2022) were available to examine the complex dynamics and interplay of ED psychopathology, objective binge eating, self‐induced vomiting, laxative use, BMI, anxiety, depression, self‐efficacy, social support, health‐related quality of life, and well‐being measured at six points for 14 months.

The objective of the present study was to explore the dynamic clustering of symptoms over the course of a low‐threshold internet‐based intervention directed at decreasing ED symptoms and increasing help‐seeking. We aimed to identify potential temporal patterns in a sample of individuals with ED symptoms. More knowledge about the co‐occurrence of symptoms in time is needed to provide personalized support in interventions such as Featback. Based on previous literature, it was expected that depressive symptoms, binge eating, and social support would change together in time and form one dimension (Dingemans et al. 2020; Fairburn et al. 2003). Furthermore, it was expected that anxiety symptoms, restrictive behaviors (like dieting and fasting), and BMI would form a dimension given the ego‐syntonic nature of restricting EDs like AN and the rewarding anxiety‐reducing effects of starving and low weight (Del Valle et al. 2017).

Furthermore, we performed directed analyses in which symptoms or attributes could be identified that exerted a significant temporal relationship on others (high out‐strength) and those symptoms or attributes that were highly influenced by others (high in‐strength). This analytical approach allows for nuanced analysis of symptom changes over time by examining how symptoms influence one another. In this context, ‘out‐strength’ refers to the extent a particular symptom influences others, while ‘in‐strength’ reflects how much a symptom is influenced by others. We hypothesized that social support would have a high out‐strength given that its baseline value was found to be a predictor for changes in ED psychopathology (Rohrbach, Fokkema, et al. 2023). We hypothesized that ED symptomatology would have a high in‐strength.

## 2 Method

### 2.1 Participants and Procedure

This study was performed as part of a randomized controlled trial (Rohrbach et al. 2019), which was preregistered in the Dutch Trial Register (NL7065) and approved by the local medical ethics committee (METC‐LDD, NL64553.058.18). The present study was preregistered at the Open Science Framework (https://doi.org/10.17605/OSF.IO/2FKW8). A comprehensive study protocol with detailed descriptions of the methods (Rohrbach et al. 2019) and the results of the RCT (Rohrbach, Dingemans, Spinhoven, et al. 2022) was published. We used the same sample in the current study. The majority of the 355 participants were recruited through Proud2Bme.nl, a Dutch e‐community for individuals with eating‐related issues. Other recruitment sources included the Featback website, a blog on a fashion and health website catering to (female) teenagers, social media, Google Ads, and the Dutch eating disorder patient organization. Eligible participants were 16 years or older and reported at least mild symptoms of an ED. They had to have a score of 52 or higher on the Weight Concerns Scale (Killen et al. 1993) or, as reported on the Short Evaluation of Eating Disorders (Bauer et al. 2005), a BMI of 18.5 or lower or at least weekly binge eating episodes or compensatory behaviors in the past 4 weeks. Individuals reporting severe ED symptoms were not excluded from the study but were advised to also seek professional help (see for more details Rohrbach et al. 2019). Those who expressed interest underwent a screening and were then asked to complete an online informed consent form and the baseline assessment (T0). Participants were randomly allocated to four conditions (during 8 weeks): (1) Featback only, (2) Featback plus weekly expert‐patient support, (3) weekly expert‐patient support only, and (4) a waiting list/care‐as‐usual control condition. Assessments were conducted at baseline (T0), post intervention (eight weeks after baseline; T1), and at 3, 6, 9, and 12 months after the post‐intervention assessment (T2, T3, T4, and T5). All assessments involved online self‐report questionnaires. Participants in one of the Featback conditions (conditions 1 and 2) received a weekly monitoring questionnaire with four questions on ED‐related symptoms. After completing the monitoring questionnaire, participants received a supportive feedback message that matched the participant's answers from a database with over 1250 different messages, written in collaboration with expert patients, scientists, and psychologists. The weekly feedback message was dependent on whether participants indicated they had improved, deteriorated, or stayed the same compared to the previous week regarding the four monitoring questions. Participants in one of the weekly expert‐patient support conditions (condition 2 and 3) were offered the option to choose between email and chat support. Chat sessions closed automatically after 20 min. For email sessions, participants were asked to send an email to their expert patient before the scheduled appointment so that the expert patient could respond during the 20‐min appointment. All participants in the four conditions were permitted to seek help from any source for their ED symptoms or other concerns. As such, the waiting list control condition (condition 4) can be considered treatment as usual. The data from all four groups were combined and analyzed as a prospective cohort study with repeated measurements to investigate the dynamics of various factors over time. This approach allows us to focus on within‐person changes and the relationships between factors across multiple time points.

### 2.2 Measurements

The outcome measures in this study were ED symptomatology (i.e., severity ED psychopathology, frequency binge eating episodes, frequency vomiting, and self‐reported BMI) evaluated using the Eating Disorder Examination Questionnaire (EDE‐Q 6.0) (Fairburn and Beglin 2008) and symptoms of anxiety and depression, which were assessed using the 4‐item self‐report Patient Health Questionnaire (PHQ‐4) (Kroenke et al. 2009). In addition, general self‐efficacy was measured with the General Self‐Efficacy Scale (GSES) (Schwarzer and Jerusalem 1995), and experienced social support was evaluated using the 12‐item Social Support List (SSL‐12‐I) (Van Eijk et al. 1994). Furthermore, health‐related quality of life was measured by means of the EQ‐5D‐5L (EuroQol Group 1990). The visual analogue scale (VAS) of the EQ‐5D‐5L was also used to record the self‐rated health of participants on a scale ranging from 100 ‘best health you can imagine’ to 0 ‘worst health you can imagine’. Well‐being was assessed with the ICEpop Capability Measure for Adults (ICECAP‐A) (Rohrbach, Dingemans, Groothuis‐Oudshoorn, et al. 2022). See for more details (Rohrbach et al. 2019). In summary, the variables (also referred to as attributes or factors in DTW) used in the analyses of the current study were: (1) BMI, (2) experienced social support, (3) self‐efficacy, (4) ED psychopathology EDE‐Q global score, (5) frequency of objective binge eating, (6) frequency of self‐induced vomiting, (7) frequency of laxatives misuse, (8) PHQ anxiety, (9) PHQ depression, (10) well‐being, (11) health‐related quality of life, (12) self‐rated health.

### 2.3 Statistical Analysis

#### 2.3.1 Missing Data Imputation

We applied multiple imputation techniques (Rubin 1987) to address missing data in the dataset, using the mice package in R (version 3.5.1). Missing data were imputed 100 times, creating 100 complete versions of the incomplete dataset. For details on the imputation procedure, see (Rohrbach, Dingemans, Spinhoven, et al. 2022). For the analyses in this study, the DTW analyses in all 100 datasets were done and thereafter averaged. For the ICECAP, EQ‐5D‐5L, and EQ‐5D‐5L VAS measures, no baseline (T0) values were collected. These values appear stable over a short period (i.e., 8 weeks) of time. Consequently, baseline values were estimated to be equal to post‐intervention (T1) values. Accordingly, this approach was validated in sensitivity analyses in a previous study (Rohrbach, Dingemans, Spinhoven, et al. 2022).

#### 2.3.2 Dynamic Time Warp Analyses

Several analyses were performed to investigate how symptom scores clustered together in individuals over the course of treatment. Attributes were first centered at the individual level to account for large between‐person differences in their mean level, before applying group‐level standardization to emphasize within‐person changes over time. Second, the group‐level mean effects of attributes over time were analyzed and plotted. Third, we conducted undirected Dynamic Time Warp (DTW) analyses to find out which attributes tended to covary in time. Fourth, we conducted directed (temporal) analyses to find out whether changes in specific attributes tended to precede similar changes in other attributes. Fifth, we examined which attributes seemed to be most influential, that is, those with a high out‐strength (i.e., temporal lead) and in‐strength (i.e., temporal lag).

Mixed models were used to estimate the average effects of the attributes measured over time. A random intercept was included for each participant to account for individual differences. Since the attributes self‐efficacy, social support, health‐related quality of life (EQ‐5D‐5L index score), well‐being (ICECAP‐A index scores), and BMI were inversely associated with other variables, we reverse‐coded these so that higher scores consistently indicated worse outcomes across all attributes.

Undirected DTW allowed identifying patterns and connections between different attributes, understanding how changes in one attribute covaried with other attribute changes within individuals. Item scores were centered within each individual and thereafter group‐level standardized before the DTW analyses. Within each participant, each attribute pair of time series data consisted of a sequence of six data points (T0–T5). We applied a Sakoe‐Chiba band constraint, creating a fixed‐width band around the main diagonal of the alignment plane in the cost matrix to ensure that alignments remained focused on temporally proximate elements (Giorgino 2009). A Sakoe‐Chiba time window size of one and a “symmetric2” step‐pattern was used. For an explanation of the analytical steps of undirected DTW analysis, see (Kopland et al. 2024; Mesbah et al. 2024; Slof‐Op 't Landt et al. 2022). The individual co‐occurrence patterns were aggregated at the group level, and hierarchical clustering was applied to identify clusters of attributes that showed similar changes over time. The results of the cluster analysis were visualized in a network plot. The networks displayed green edges where items had similar valences (e.g., depression and anxiety) and red edges where items had opposite valences (e.g., well‐being and depression).

In the directed DTW analysis, stretching (i.e., warping) was permitted in only one direction, only forward in time (consistent with a temporal lag‐1 approach), resulting in directed distances. This directed approach helps determine whether shifts in one attribute tended to occur before or after changes in another attribute, to find the network of positive feedback and feedforward interactions among the attributes. We conducted directed (temporal) DTW analysis employing an asymmetric band window type of size 1. This means the DTW procedure aligns with either the current time point (lag‐0) or the subsequent time point (lag‐1) of the corresponding data point in the other time series. Only when the directed distance at the group level is significantly larger than zero (*α* = 0.05), an arrow is shown in the directed network plot. We conducted one test for each node as the main outcome, which resulted in 12 tests in total. The present study was a proofofprinciple designed to demonstrate how time series data can be analyzed differently in the field of ED research. For a more detailed explanation of the analytical steps of directed DTW analysis, see (Kopland et al. 2024; Mesbah et al. 2024; Van Der Does et al. 2023).

Finally, the standardized out‐strength (i.e., temporal lead) and in‐strength (i.e., temporal lag) centrality values on the group level were assessed with the 95% confidence intervals. Attributes with a significant out‐strength indicate that fluctuations in these attributes tended to precede those of all other attributes rather than vice versa, whereas changes in attributes with significant in‐strength tended to follow similar changes in other attributes. The “dtw” (version 1.23–1), “parallelDist” (version 0.2.6), “lme4” (version 1.1–35.1) and “qgraph” (version 1.9.8) packages for the R statistical software were used.

## 3 Results

### 3.1 Participants

A total of 355 participants completed both the informed consent process and the baseline assessment, making them eligible for analysis. Table 1 presents the baseline characteristics of the participants. The retention rates for participants were 100% at baseline (T0), 78.9% at post‐intervention (8 weeks; T1), and 71.0%, 68.7%, 65.6%, and 68.2% at 3, 6, 9, and 12‐month follow‐ups (T2–T5), respectively. There were no significant differences in dropout rates observed between conditions at either the post‐intervention assessment (χ2(3) = 3.99, *p* = 0.26) or the 12‐month follow‐up (χ2(3) = 4.90, *p* = 0.18). Additionally, no distinctions were noted in the discontinuation of the intervention across the three active intervention groups (χ2(2) = 1.24, *p* = 0.54).

**TABLE 1:** Clinical and demographical characteristics of the total sample (*N* = 355).

Characteristics	Total sample (*N* = 355)
Gender	
Female (%)	343 (96.7)
Male (%)	9 (2.5)
Other (%)	3 (0.8)
Nationality	
Dutch (%)	319 (89.9)
Belgian (%)	32 (9.0)
Other (%)	4 (1.1)
Education	
Low (%)	47 (13.3)
Middle (%)	133 (37.6)
High (%)	174 (49.2)
Treatment history for ED	
Yes (%)	202 (56.9)
No (%)	153 (43.1)
Marital status	
Married/living together (%)	98 (27.6)
Living alone (%)	250 (70.4)
Divorced (%)	6 (1.6)
Widow (%)	1 (0.2)
Age [Years]	27.8 (10.8)
BMI Years with ED	22.1 (7.0) 10.1 (9.7)
EDE‐Q (range 0–6)	4.1 (1.0)
Frequency binge eating, past 28 days	7.8 (8.6)
Frequency vomiting, past 28 days	4.3 (9.9)
Frequency laxatives use, past 28 days	2.9 (9.5)
PHQ‐4 (range 0–12)	7.8 (3.2)
GSES (range 10–40)	26.12 (5.6)
SSL‐12 (range 12–48)	30.0 (7.0)
RSES (range 10–40)	20.5 (5.3)
Motivation to change (1–100)	22.0 (4.5)
Internet usage [hours per day]	3.8 (2.5)

*Note*: Data are presented as means (SD) unless indicated otherwise.

Abbreviations: ED = Eating Disorder; EDE‐Q = Eating Disorder Examination Questionnaire; GSES = General Self‐Efficacy Scale; PHQ‐4 = 4‐item Patient Health Questionnaire; RSES = Rosenberg Self‐Esteem Scale; SD = standard deviation; SSL‐12 = 12‐item Social Support List.

### 3.2 Symptom Network of Co‐Occurring Attributes: Undirected Dynamic Time Warp

**FIGURE 1:**
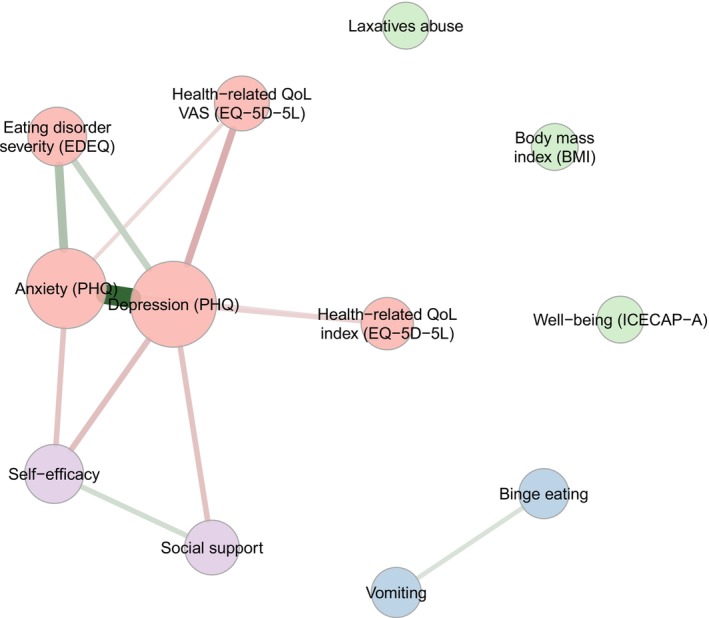
Undirected network. N.B. The edges represent the statistically significant influences between the attributes and were color‐coded: Green indicating a positive relationship and red indicating a negative one. The thickness of the edges indicates the strength of the connection.

**FIGURE 2:**
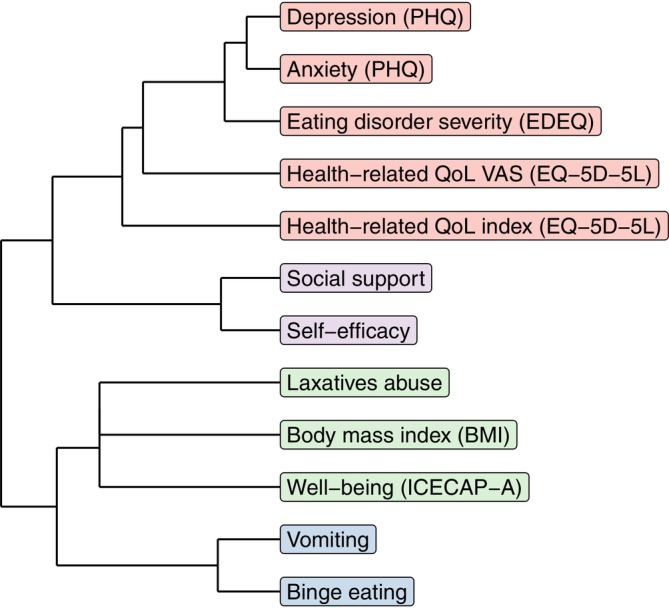
Dendrogram undirected network.

**FIGURE 3:**
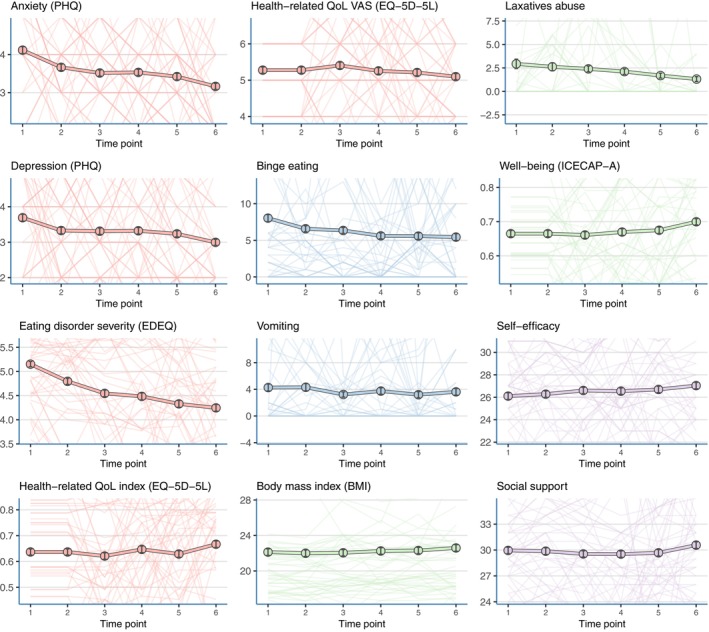
Mean item scores across six timepoints.

Figure 1 portrays the undirected network, which addresses the aim regarding the similarities in the change patterns of attributes across the four conditions and across the six assessments (T0‐T5). We explored which factors collectively change in time and form dimensions. Hierarchical cluster analysis indicated an optimum of four dimensions which were color coded (see Figures 1 and 2). The edges represent the statistically significant influences between the attributes and were color‐coded: green indicating a positive relationship and red indicating a negative one. The thickness of the edges indicates the strength of the connection. The dimension were named: (1) psychological health (containing the attributes anxiety, depression, health‐related quality of life and self‐rated health, and ED severity; red color), (2) psychosocial resilience (containing the attributes social support and self‐efficacy; purple color), 3) disordered eating behaviors (containing the attributes self‐induced vomiting and binge eating; blue color) and finally, dimension (4) weight management, which appeared to be the least well‐defined cluster (containing the attributes: laxatives abuse, BMI, and well‐being; green color). The means of the attributes over the six time points is reported in Figure 3.

### 3.3 Symptom Network of Temporal Effects: Directed Dynamic Time Warp

**FIGURE 4:**
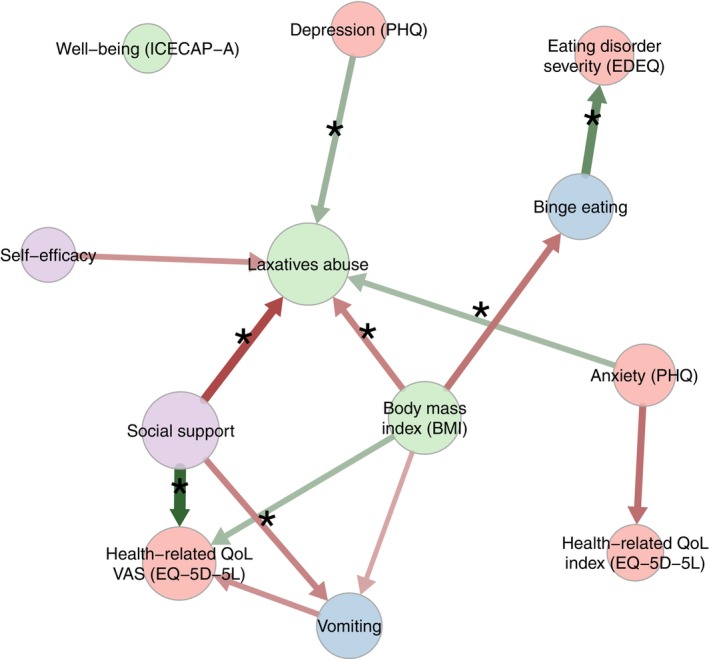
Directed network. N.B. The edges represent the statistically significant influences between the attributes and were color‐coded: Green indicating a positive relationship and red indicating a negative one. The thickness of the edges indicates the strength of the connection.

**FIGURE 5:**
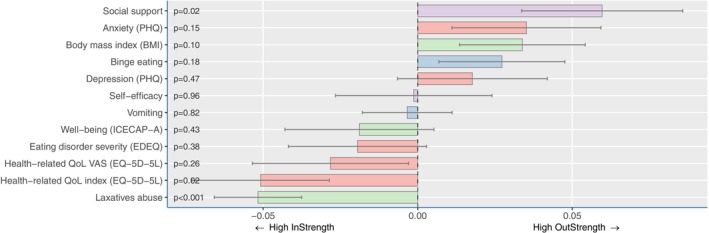
Directed network in‐ and outstrengths.

Figure 4 displays the directed network, where attributes are delineated within the overarching network. Some attributes exhibited a pattern of preceding and predicting other attributes (high out‐strength or temporal lead), while others showed a significant susceptibility to influence from other attributes (high in‐strength or temporal lag). The arrows represent the statistically significant influences between the attributes and are color‐coded: green indicating a positive relationship and red indicating a negative one. The thickness of the edges indicates the strength of the connection (all edges, out‐strength and in‐strength, are reported in a forest plot in Figure 5). The analyses indicated that social support and anxiety were attributes with high out‐strength. These attributes were influential in the network, and changes in these attributes often preceded changes in other attributes. Laxative abuse and health‐related quality of life had high in‐strength, suggesting that they are influenced by other attributes.

## 4 Discussion

The aim of the present study was to gain deeper insights into personal dynamics and to enhance our understanding of the processes of change and the interaction of symptoms during and after an ED intervention. The group‐level analysis and undirected DTW analysis revealed four clusters in which the symptom severity covaried and changed similarly over time. The strongest cluster, named “psychological health,” involved the attributes anxiety, depression, health‐related quality of life and self‐rated health, and ED psychopathology (i.e., ED attitudes), followed by the cluster named “psychosocial resilience,” containing the attributes social support and self‐efficacy, and the cluster “disordered eating behaviors.” The fourth cluster, named “weight management,” appeared less strong, indicating that the patterns in which these attributes changed were more independent than the changes of attributes in other clusters. The clusters were partly in line with expectations, in that anxiety and ED symptomatology clustered together. In contrast to the hypotheses, social support, depression, and binge eating did not have similar temporal patterns. However, while the composition of the clusters deviates from the hypotheses, it is compatible with earlier research. Mainly, the strongest cluster, ‘psychological health’, underscores the intricate interplay and co‐occurrence of (mental) health dimensions. Anxiety and depression are well‐established comorbid conditions, often co‐occurring with ED symptoms and exacerbating each other (Filipponi et al. 2022; Hambleton et al. 2022). The association with health‐related quality of life and self‐rated health adds another layer of complexity to this covariation. Health‐related quality of life and self‐rated health encompass various domains, ranging from mobility, self‐care, usual activities, pain/discomfort, to anxiety/depression. Poor psychological health appears to be associated with a negative self‐assessment of one's overall health, reflecting a bidirectional interplay between psychological distress and feeling healthy.

Directed analyses revealed that social support and anxiety were the most central attributes with the highest out‐strength, while laxative use and well‐being were among the most influenced (i.e., highest in‐strength). These results were partly in line with expectations, where especially social support was hypothesized to have a high out‐strength. Indeed, several studies indicated that there is an association between an increase in social support and the recovery of mental disorders (Li et al. 2023; Monistrol‐Mula et al. 2022) which also applies to EDs (Geller et al. 2017; Rohrbach, Fokkema, et al. 2023). As expected, the present study showed that the enhancement of social support preceded the improvement of symptoms. Encouraging sharing with and involving loved ones seems to be an essential part of the recovery process (Linville et al. 2012). The improvement of social support should be prioritized early in the treatment process. ED symptomatology was expected to have a high in‐strength, but this was not found.

With regard to anxiety, this symptom stands out as a key factor in EDs that exacerbates the persistence of ED behaviors and psychopathology (Aldao et al. 2010; Fitzpatrick et al. 2019; Sander et al. 2021). Individuals with EDs engage in behaviors such as dieting (Lavender et al. 2013), purging (Levinson et al. 2017), and binge eating (Rosenbaum and White 2015) to cope with unwanted feelings of anxiety (Polivy et al. 1994) triggered by stressful situations. As expected, the finding in the present study that anxiety is a central attribute with a high out‐strength and that laxative abuse is an attribute with a high in‐strength is in line with these studies. This suggests that when seeking support or treatment is successful in reducing (ED‐related) anxiety symptoms, it can help individuals no longer feel the need to engage in specific ED behaviors, such as using laxatives. These factors are addressed in the feedback messages as well as in the chat and emails of the expert patients of the online interventions. Arguably, a similar mechanism applies to well‐being. Well‐being was found to have a high in‐strength, suggesting that improvement after a change in other attributes such as social support, a healthy weight, or anxiety symptoms is instigated. In their study, Kopland et al. (2025) found comparable results using the same network techniques. Results indicated that a change in hopelessness, worrying, and restlessness (which are symptoms of anxiety and depression) drives improvement in ED behaviors and self‐compassion. This is similar to how anxiety was found to precede improvement in ED‐related symptoms and well‐being in the current study. Indeed, well‐being and self‐compassion appear to be distinct but closely related concepts. Contrary to expectations, no conclusive evidence was found to suggest that ED symptomatology had a high in‐strength. While ED behaviors (such as laxative abuse and vomiting) and ED attitudes (including ED severity) were identified as high in‐strength factors, only the former proved to be significant. One possible explanation for this finding is that the 8‐week, low‐intensity anonymous online intervention, designed to serve as an initial step toward recovery, may have had only a limited impact on ED symptomatology.

### 4.1 Clinical Implications

From a clinical perspective, our findings highlight the importance of adopting a holistic approach to mental health assessment and interventions. Rather than treating symptoms in isolation, clinicians should consider the interconnectedness of various mental health dimensions and target underlying mechanisms that contribute to their co‐occurrence. Interventions for EDs often already target multiple symptoms. Indeed, effectiveness studies for cognitive behavioral therapy (CBT) for EDs often find improvements in ED symptoms, as well as other areas such as general psychopathology (Dalle Grave et al. 2013; Trottier et al. 2022) and self‐esteem (de Jong et al. 2020). Comparable results are expected for digital interventions (Melioli et al. 2016) as these are often based on CBT principles. Additionally, treatment guidelines for EDs involve intervention in different life domains, including ED behaviors, strengthening the social network, and self‐esteem (Netwerk Kwaliteitsontwikkeling GGz 2018). Increasingly, loved ones are an important part of the recovery process (Linville et al. 2012; Rienecke 2017), indicating that the importance of social support is acknowledged.

However, these guidelines and interventions are mostly based on group research and clinical experience, while specific knowledge on which factors to target when in the treatment is still in its infancy. Current evidence‐based ED treatments target multiple symptoms, while this study highlights the need for greater personalization and to address symptom‐specific interactions like anxiety and ED behaviors. Addressing and enhancing social support appear to be important elements in treatment and important catalysts for change. Internet interventions may be especially fitting tools to provide social support for individuals who find the step toward face‐to‐face treatment too daunting, often due to the shame and stigma associated with EDs and barriers to accessing treatment, skills training, or support (Aardoom et al. 2016b). Indeed, patients and carers found online programs supportive and indicated that they were more likely to engage in online support than access face‐to‐face treatment or support as a first step (Yim et al. 2021). Online individual support by means of chat or mail can also provide the possibility to address the specific attributes most central to that specific individual and stimulate seeking social support (Rohrbach, Dingemans, Spinhoven, et al. 2022). Since social support appears to be crucial to recovery from an ED, incorporating tools that enhance social support in online interventions is warranted, for example in the form of a forum or the possibility of receiving counseling from peers, expert patients, or psychologists by means of chat or email (Rohrbach, Dingemans, Spinhoven, et al. 2022; Yim et al. 2021).

### 4.2 Strengths and Limitations

A strength of the present study is that this relatively new method of temporal network data analysis yields insights into the central factors that drive others. Second, we included a relatively large sample measured at six different time points over 14 months. The present study should also be seen considering several limitations. First, the results may not be generalized to contexts outside the field of anonymous internet interventions. Relatedly, the analyses were performed in the context of a RCT, and other clusters may be found in other designs, such as observational studies. Secondly, most of our sample was female and white, making it hard to generalize results to a more diverse population. Thirdly, baseline values of quality of life (EQ‐5D‐5L) and well‐being (ICECAP‐A) questionnaires were missing. Although a sensitivity analysis with estimated baseline scores produced similar results (see for details Rohrbach, Dingemans, et al. 2023), the missing values may have led to a slight underestimation of these variables at baseline. Fourth, the between‐person variability in observed symptom dynamics was substantial, as evidenced by the wide confidence intervals in Figure 5. The multi‐interpretability of several items of the EDEQ could have increased the difference between persons for this measure. This variability underscores the need for personalized approaches, which could be achieved with more frequent measurements within an individual. Such an approach would allow for the identification of influential symptoms at an individual level, enabling targeted and tailored treatment strategies. This aligns with the insights provided by Hekler et al. (2019), who emphasize the value of small data approaches in capturing idiosyncratic phenomena. A final limitation is the use of self‐report measures, which are subject to socially acceptable answers, recall bias, and misinterpretation. However, in the present sample, using self‐report measures was required to ensure the low‐threshold character of the intervention and the anonymity of participants.

## 5 Conclusion

In conclusion, the results suggest that patterns of symptoms and other factors can be identified that tend to covary and evolve similarly over time. The cluster labeled psychological health, with anxiety, depression, health‐related quality of life, self‐rated health, and ED psychopathology, emerged as the strongest cluster. This highlights the interconnectedness of various mental health dimensions, which may be important to consider in treatment. Rather than addressing symptoms in isolation, it is warranted to target the underlying mechanisms that contribute to their co‐occurrence. Additionally, the present study suggests that increasing social support may be an important factor to address early, as it was found to drive changes in other symptoms and factors.

## Author Contributions

**A. E. Dingemans:** conceptualization, funding acquisition, investigation, project administration, writing – original draft, writing – review and editing. **E. J. Giltay:** conceptualization, data curation, formal analysis, investigation, methodology, writing – original draft, writing – review and editing. **P. J. Rohrbach:** data curation, writing – original draft, writing – review and editing. **E. F. van Furth:** funding acquisition, writing – review and editing. **M. C. T. Slof‐Op ’t Landt:** conceptualization, writing – original draft, writing – review and editing.

## Ethics Statement

Ethical approval for this study was obtained from the Leiden University Medical Centre Ethical Committee (CME LUMC Leiden, reference number NL64553.058.18). All participants gave online informed consent for participating in the study.

## Conflicts of Interest

The authors declare no conflicts of interest.

## Data Availability

The datasets generated and analyzed for the current study are available from the corresponding author on reasonable request and are included in the DANS repository.
